# Supplementary material for: Adaptive optical waveguide system for large-area and overheating-preventing phototherapy in deep tissue
Source: Nat Commun. 2026 Feb 28;17:3308. doi: 10.1038/s41467-026-69759-5 (PMC13066437; doi:10.1038/s41467-026-69759-5)
Supplement: Supplementary file 1 — Supplementary Information [file 41467_2026_69759_MOESM1_ESM.pdf]

# Supplementary information

# Adaptive optical waveguide system for large-area and overheating-preventing phototherapy in deep tissue

Zhenhao Wang[1], Zhaoxiang Yang[1], Yingchao Ma[1], Shanzhi Lyu[1], Xiaohua Hu[2], Yapei Wang[1]*

[1] School of Chemistry and Life Resources, Renmin University of China, Beijing, 100872, China

[2] Department of Burns and Plastic Surgery, Beijing Jishuitan Hospital, Capital Medical University, Beijing, 100035, China

**1. Supplementary Discussion and Analysis** 3-11

1.1 Simulation analysis 3-4

1.2 Calculation of photothermal conversion efficiency 5-6

1.3 The minimum deviation angle method 7-9

1.4 Analysis of divergence angle and optical transmission efficiency 10-11

**2. Supplementary Tables and Figures** 12-40

2.1 Supplementary Tables 12-15

2.2 Supplementary Figures 16-47

**3. References for Supplementary Information** 48-49

# 1. Supplementary Discussion and Analysis

## 1.1 Simulation analysis

To assess the performance of AOWS in enabling deeper phototherapy while protecting tissues under photothermal conditions, two unsteady-state heat transfer models were developed using finite element analysis (FEA). These models were used to predict temperature distributions. Simulations were performed using Ansys software, and the initial temperature of the entire simulated object was set as 34 °C. The results reflect the subcutaneous temperature of animals (as shown in Fig. 6b).

The first FEA model was designed to predict the influence of varying core temperatures of photothermal therapy materials on treatment outcomes. The abnormal tissue was modeled as a sphere with a radius of 6.2 mm, corresponding to a volume of 1000 $mm^3$. The photothermal therapy material was represented as a smaller sphere with a radius of 1.0 mm. Core temperatures were set as 60 °C, 65 °C, 70 °C, 75 °C, 80 °C, 85 °C, and 90 °C, respectively. The ambient temperature was held constant at 25°C, and the simulation duration was 60 s. After performing a grid independence test, a total of 105005 cells were used for this FEA model (Supplementary Fig. 1). The results, presented in Supplementary Figs. 2 and 3, indicated that only core temperatures exceeding 80 °C ensured effective photothermal therapy for abnormal tissue. However, these temperatures also reached the threshold for tissue carbonization.

The other FEA models were employed to predict the temperature distribution within abnormal tissue, corresponding to the conditions observed in animal experiments. In these simulations, we modeled two scenarios: one using AOWS for

photothermal therapy and the other using OWS. The photothermal therapy material was represented as a cylinder with a radius of 2.5 mm and a thickness of 1 mm. The distance between the photothermal therapy material and the optical fiber outlet was set to 4.0 mm. The temperature changes in the photothermal therapy materials for both models aligned with experimental results (Fig. 6b). The ambient temperature was maintained at 25 °C, and the simulation was run for 2000 s. Following a grid independence test, a total of 103978 cells were used for this FEA model (Supplementary Fig. 31). The results are presented in Fig. 6d and Supplementary Fig. 26. The comparison between experimental and simulated results showed strong agreement (Supplementary Fig. 32).

## 1.2 Calculation of photothermal conversion efficiency

The photothermal conversion efficiency ($\eta_{PT}$) of the PPy NPs was determined following our previously reported methodology[1]. First, the UV-Vis-NIR absorption spectrum of a 0.08 mg/mL PPy NPs suspension was recorded to obtain the absorbance at 808 nm (Supplementary Fig. 25b). Next, 1.0 g of this suspension was placed in a quartz cuvette and irradiated at five different power densities. Each irradiation lasted two minutes after the sample had equilibrated to its initial temperature, and five consecutive heating-cooling cycles were performed at each power setting to evaluate reproducibility (Supplementary Fig. 25c, d). The typical $\eta_{PT}$ at a fixed power was calculated according to Equation. S1:

$$\eta_{PT} = \frac{hA\Delta T_{max}}{I(1-10^{-A_{\lambda}})} \quad \text{(S1)}$$

Where $h$ is the heat transfer coefficient, $A$ is the surface area of the system, $\Delta T_{max}$ is the temperature difference between the maximum temperature and initial temperature, $I$ is the light power, $A_{\lambda}$ is the absorbance at wavelength $\lambda$. In this equation, only $hA$ is unknown. In order to get $hA$, $\theta$ defined as the ratio of $\Delta T$ to $\Delta T_{max}$ was introduced and the value of $hA$ is derived according to Equation S2:

$$t = -\frac{\sum_i m_i C_{p,i}}{hA} ln\,\theta \quad \text{(S2)}$$

Therefore, $hA$ can be determined by the linear time data from the cooling period $t$ vs $-\ln\theta$. The weight (m) of PPy NPs solution was 1.0 g and specific heat ($C_p$) was determined to be 4.20 J/(g·°C). Substituting $hA$ value into Equation S1, the photothermal conversion efficiency ($\eta_{PT}$) could be calculated.

We selected the second heating-cooling cycle at each irradiation power to calculate the corresponding photothermal conversion efficiency ($\eta_{PT,I}$). All parameters used in these calculations are detailed in Supplementary Table 1. The mean photothermal conversion efficiency of the PPy NPs was determined to be 42.27 % ± 2.92 %, which agrees well with literature values[2].

### 1.3 The minimum deviation angle method

To ensure maximum accuracy in our optical simulations, we obtained a precise measurement of the refractive index of the aqueous ionic liquid at 532 nm (Supplementary Fig. 21). Because the Abbe refractometer only provides data at 589.3 nm, we instead employed the minimum deviation angle method[3].

In this approach, a hollow triangular prism (apex angle $\alpha$) is filled with liquid and illuminated by a 532 nm laser along the DE face; the emergent beam exits the FG face. We define the total angular deviation $\delta$ between the incident ray DE and the emergent ray FG. From simple geometry:

$$\delta = \angle 1 + \angle 2 = (i_1 - i_2) + (i_1{}' - i_2{}') = (i_1 + i_1{}') - (i_2 + i_2{}') \quad \text{(S3)}$$

$$i_2 + i_2{}' + (\pi - \alpha) = \pi \quad \text{(S4)}$$

Equations S3 and S4 yield:

$$\alpha = i_2 + i_2{}' \quad \text{(S5)}$$

$$\delta = i_1 + i_1{}' - \alpha \quad \text{(S6)}$$

By Snell's law at faces AB and AC:

$$sini_1 = nsini_2 \quad \text{(S7)}$$

$$sini_1{}' = nsini_2{}' \quad \text{(S8)}$$

For a certain value of $i_1$, $\delta$ has a minimum value of $\delta_{min}$ called the minimum angle of deflection, which can be used for the measurement of the refractive index of transparent materials. At the minimum-deviation condition, $\delta$ attains its minimum $\delta_{min}$, when

$$\frac{d\delta}{di_1} = 1 + \frac{di_1{}'}{di_1} = 0 \quad \text{(S9)}$$

$$\frac{di_1{}'}{di_1} = -1 \quad \text{(S10)}$$

Differentiate Equation S5 with respect to $i_2'$ gives:

$$\frac{di_2}{di_2'} = -1 \tag{S11}$$

and differentiating Snell's law:

$$cosi_1 di_1 = ncosi_2 di_2 \tag{S12}$$

$$cosi_1' di_1' = ncosi_2' di_2' \tag{S13}$$

Dividing S12 by S13 yields:

$$\frac{cosi_1}{cosi_2} = \frac{cosi_1'}{cosi_2'} \tag{S14}$$

Squaring and substituting from S7 and S8 gives:

$$\frac{1-sin^2 i_1}{n^2-sin^2 i_1} = \frac{1-sin^2 i_1'}{n^2-sin^2 i_1'} \tag{S15}$$

which implies

$$i_1 = i_1' \text{ and } i_2 = i_2' \tag{S16}$$

Hence, at minimum deviation:

$$i_2 = \frac{\alpha}{2} \tag{S17}$$

$$i_1 = \frac{1}{2}(\delta_{min} + \alpha) \tag{S18}$$

Substituting S17 and S18 into Snell's law at face AB leads to

$$sin\frac{\delta_{min}+\alpha}{2} = nsin\frac{\alpha}{2} \tag{S19}$$

from which the refractive index is determined as

$$n = \frac{sin\frac{\delta_{min}+\alpha}{2}}{sin\frac{\alpha}{2}} \tag{S20}$$

Specifically, a custom hollow triangular prism with a right-triangle base and an apex angle $\alpha$ of 60° was fabricated and filled with the aqueous ionic liquid solution. Under these geometric conditions, the minimum deviation angle is achieved when the incident angle $i_2$ equals 30°, corresponding to the scenario in which the emergent ray

EF is parallel to the base BC of the triangle. The minimum angle of deflection $\delta_{min}$ was measured using a high-precision universal angle protractor. The refractive index at 532 nm ($n_{532}$) was then calculated using Equation S20.

Since light at 808 nm lies outside the visible spectrum and is unsuitable for the minimum deviation method, the refractive index at 589.3 nm ($n_{589.3}$) was measured using an Abbe refractometer. Subsequently, the refractive index at 808 nm ($n_{808}$) was estimated by applying the two-wavelength Cauchy dispersion relation to extrapolate from the measured values at 532 nm and 589.3 nm.

$$n = A + \frac{B}{\lambda^2} \tag{S21}$$

Both A and B are constants. All parameters used in these calculations are detailed in Supplementary Table 2. And the refractive indices at different wavelengths for silicone rubber and nanotape were obtained from the suppliers.

### 1.4 Analysis of divergence angle and optical transmission efficiency

As described in the main text, the transparent elastomer encapsulation surrounding the fiber optic tip can be deformed by varying the volume of injected ionic liquid solution into convex, plain or concave shapes, with curvature precisely tuned to achieve specific divergence angles. Using the refractive index values in Table 2, we performed RSoft simulations of light propagation at 532 nm for each deformation state of the AOWS head (Fig. 4e and Supplementary Fig. 23a). These simulations demonstrate that the emission angle can be actively modulated by changing the head geometry without compromising overall optical transmission efficiency. We defined the divergence angle as the angle between the emitted beam and the Z axis (positive for divergence, negative for convergence) and found it to vary from -15.13° to +24.61° in simulation, which is fully consistent with the subsequent experimental results (Supplementary Fig. 23b, c).

To validate these results experimentally, we measured both the divergence angle and the transmitted optical power under different outlet configurations. In the divergence state, the divergence angle was calculated as

$$Divergence\ Angle = tan^{-1}\frac{D_1 - D_0}{2d} \quad \text{(S22)}$$

and in the convergence state as:

$$Divergence\ Angle = -\,tan^{-1}\frac{D_1 + D_0}{2d} \quad \text{(S23)}$$

where $D_0$ is the diameter of the spot at the fiber head, $D_1$ is the diameter of the spot on the light screen, and $d$ is the distance from the fiber head to the light screen. All parameters used in these calculations at 532 nm are listed in Supplementary Table 3. The experimentally measured divergence angles ranged from -15.38 ± 0.94° to

+25.63 ± 1.51°, substantially exceeding the range offered by standard commercial optical fibers. Importantly, the optical output power did not show significant variation across different divergence angles, confirming that beam-angle modulation does not compromise power delivery. As shown in Fig. 4d, the concave cap produced a larger illumination area compared with planar or convex configurations. This capability suggests that AOWS can be configured to deliver a desired illumination profile while remaining fixed during interventional phototherapy.

We then performed the same set of RSoft simulations and experimental measurements at 808 nm (Supplementary Fig. 24). The overall trends paralleled those observed at 532 nm: both simulated and measured divergence angles varied with head geometry, while optical transmission efficiency remained effectively constant across all configurations. The range of divergence angles at 808 nm was slightly reduced compared with 532 nm, owing to the lower refractive index of the ionic liquid at this wavelength (simulation: -15.37° to +21.10°; experiment: -14.31° ± 0.60° to +23.99° ± 1.00°, Supplementry Table 4). The precise control of the divergence angle in AOWS enables closer placement of the fiber tip to the target tissue, thereby enhancing negative-feedback regulation and minimizing thermal injury to surrounding healthy tissue.

## 2. Supplementary Tables and Figures

### 2.1 Supplementary Tables

**Supplementary Table 1**. Summary of each parameter of photothermal conversion efficiency.

| I/W | $A_{808}$ | $\triangle T_{max}$/°C | $\frac{\sum_i m_i C_{p,i}}{hA}$/s | $\sum_i m_i C_{p,i}$/J·°C$^{-1}$ | $hA$/W·°C$^{-1}$ | $\eta_{PT,I}$ | $\overline{\eta_{PT}}$ | S.D. |
|---|---|---|---|---|---|---|---|---|
| 0.2 | | 2.1 | 131.9953 | | 0.0318 | 40.98% | | |
| 0.4 | | 4.1 | 123.4600 | | 0.0340 | 42.77% | | |
| 0.6 | 0.7334 | 6.6 | 135.4013 | 4.20 | 0.0310 | 41.85% | 42.27% | 2.92% |
| 0.8 | | 8.2 | 112.7826 | | 0.0372 | 46.82% | | |
| 1.0 | | 10.0 | 132.3584 | | 0.0317 | 38.92% | | |

**Supplementary Table 2**. Summary of each parameter of refractive index measurement.

| | $\boldsymbol{\alpha}$/° | $\boldsymbol{\delta_{min}}$/° | $\boldsymbol{n_{532}}$ | $\boldsymbol{n_{589.3}}$ | $\boldsymbol{A}$ | $\boldsymbol{B}$/nm² | $\boldsymbol{n_{808}}$ |
|---|---|---|---|---|---|---|---|
| IL aq | 60.00 | 28.57 | 1.3964 | 1.3950 | 1.3888 | 2141.6463 | 1.3921 |
| Silicone rubber | -- | -- | 1.42 | -- | -- | -- | 1.41 |
| Nanotape | -- | -- | 1.41 | -- | -- | -- | 1.40 |

**Supplementary Table 3**. Summary of each parameter of divergence angles at 532 nm.

| Injection Volume/mL | $D_0$/cm | $d$/cm | $\overline{D_1}$/cm | S.D./cm | $\overline{Divergence\ Angle}$/° | S.D./° |
|---|---|---|---|---|---|---|
| -0.04 | | | 2.22 | 0.13 | 25.63 | 1.51 |
| -0.02 | | | 1.00 | 0.12 | 9.92 | 1.69 |
| 0.00 | 0.30 | 2.00 | 0.62 | 0.08 | 4.572 | 1.19 |
| 0.02 | | | 0.46 | 0.05 | -10.76 | 0.76 |
| 0.04 | | | 0.80 | 0.07 | -15.38 | 0.94 |

**Supplementary Table 4**. Summary of each parameter of divergence angles at 808 nm.

| Injection Volume/mL | $D_0$/cm | $d$/cm | $\overline{D_1}$/cm | S.D./cm | $\overline{Divergence\ Angle}$/° | S.D./° |
|---|---|---|---|---|---|---|
| -0.04 | | | 2.08 | 0.08 | 23.99 | 1.00 |
| -0.02 | | | 0.94 | 0.11 | 9.09 | 1.59 |
| 0.00 | 0.30 | 2.00 | 0.54 | 0.05 | 3.43 | 0.78 |
| 0.02 | | | 0.42 | 0.08 | -10.20 | 1.16 |
| 0.04 | | | 0.70 | 0.04 | -14.308 | 0.60 |

## 2.2 Supplementary Figures

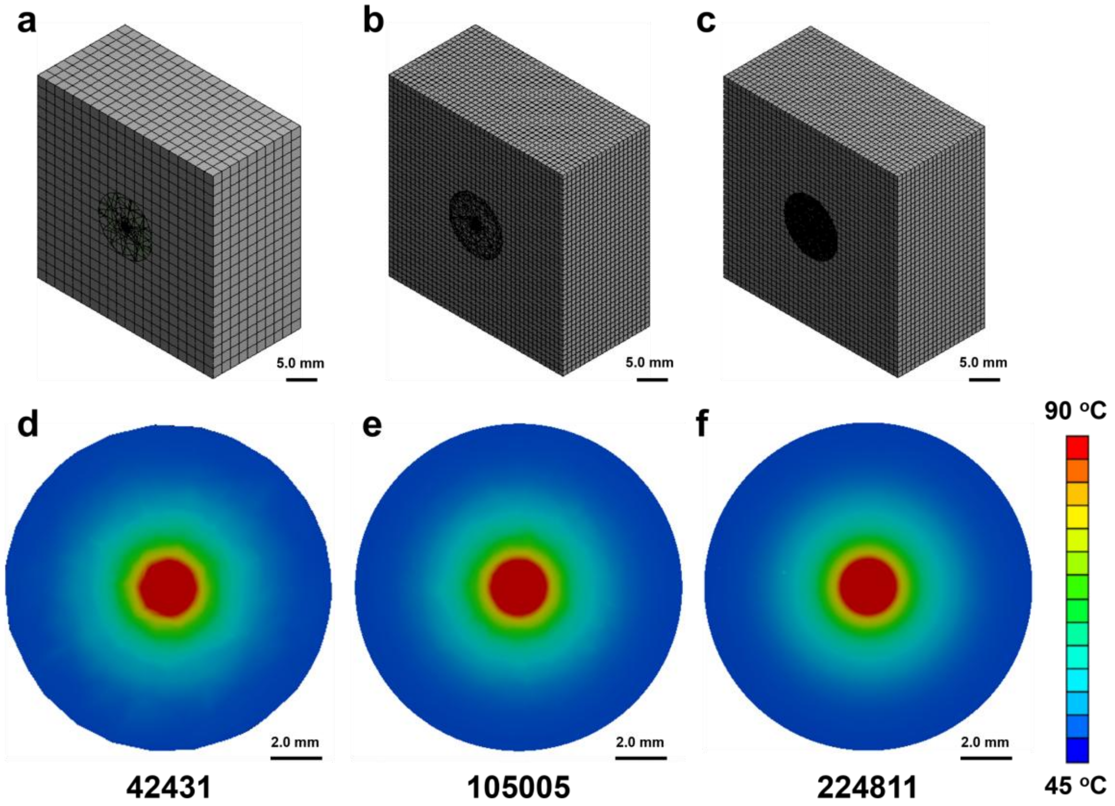


**Supplementary Fig. 1. The grid independence test for 3D temperature distribution simulation of a 1000 mm$^3$ lesion treated with PTT.** The cubic was divided into (**a** and **d**) 42431 units, (**b** and **e**) 105005 units and (**c** and **f**) 224811 units to perform the temperature distribution simulation. The consistent temperature distribution in **e** and **f** indicated the simulation was grid-independent.

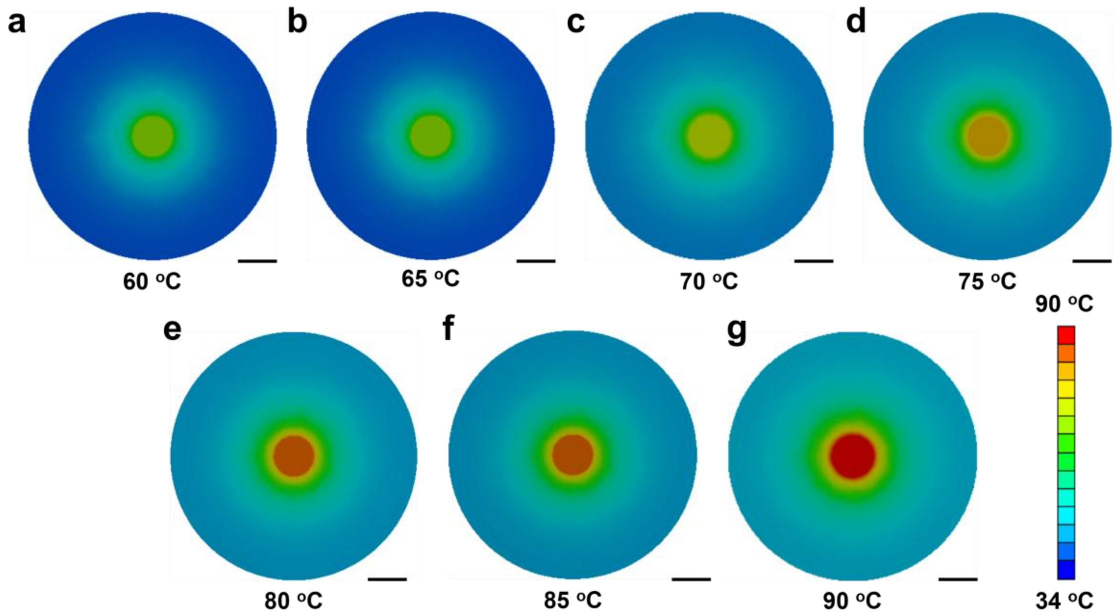


**Supplementary Fig. 2. Three-dimensional temperature distribution simulation results for a 1000 mm$^3$ lesion treated with PTT.** The core temperatures were **a,** 60 °C, **b,** 65 °C, **c,** 70 °C, **d,** 75 °C, **e,** 80 °C, **f,** 85 °C and **g,** 90 °C. Scale bars: 2.0 mm.

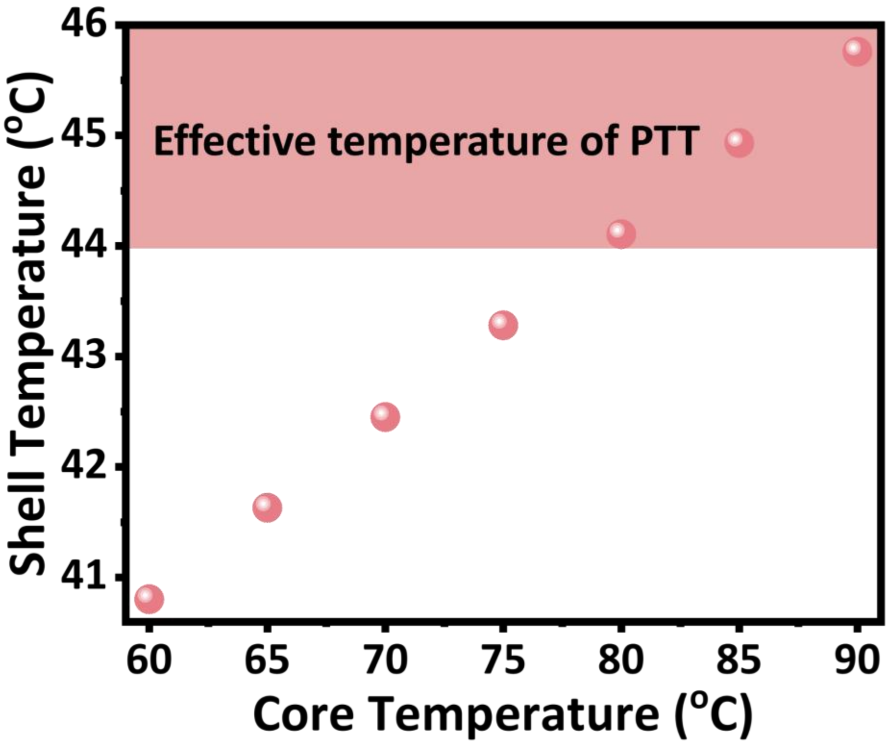


**Supplementary Fig. 3. Results of 3D temperature distribution simulations of the variation of a 1000 mm$^3$ lesion shell temperature with core temperature.**

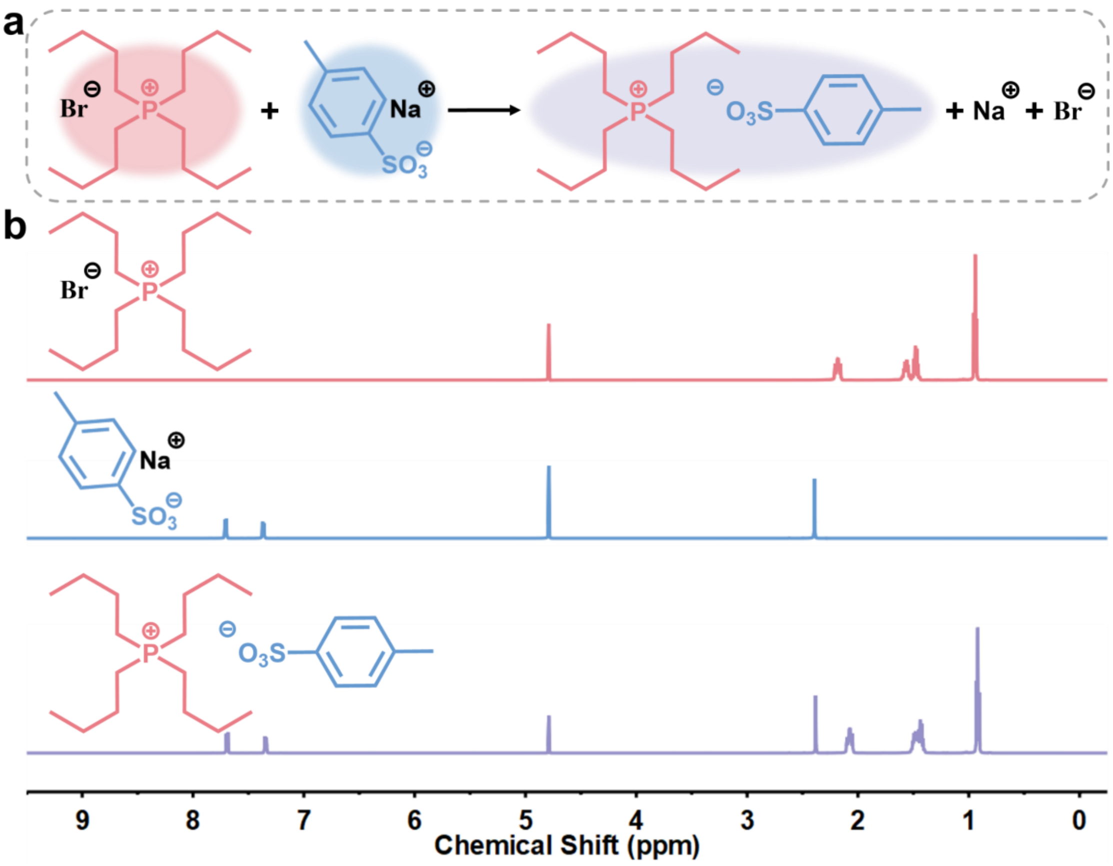


**Supplementary Fig. 4. Synthesis and characterization of [$P_{4444}$][TsO]. a,** Synthesis process of [$P_{4444}$][TsO]; **b,** $^{1}$H NMR spectra of [$P_{4444}$]Br, Na[TsO] and [$P_{4444}$][TsO].

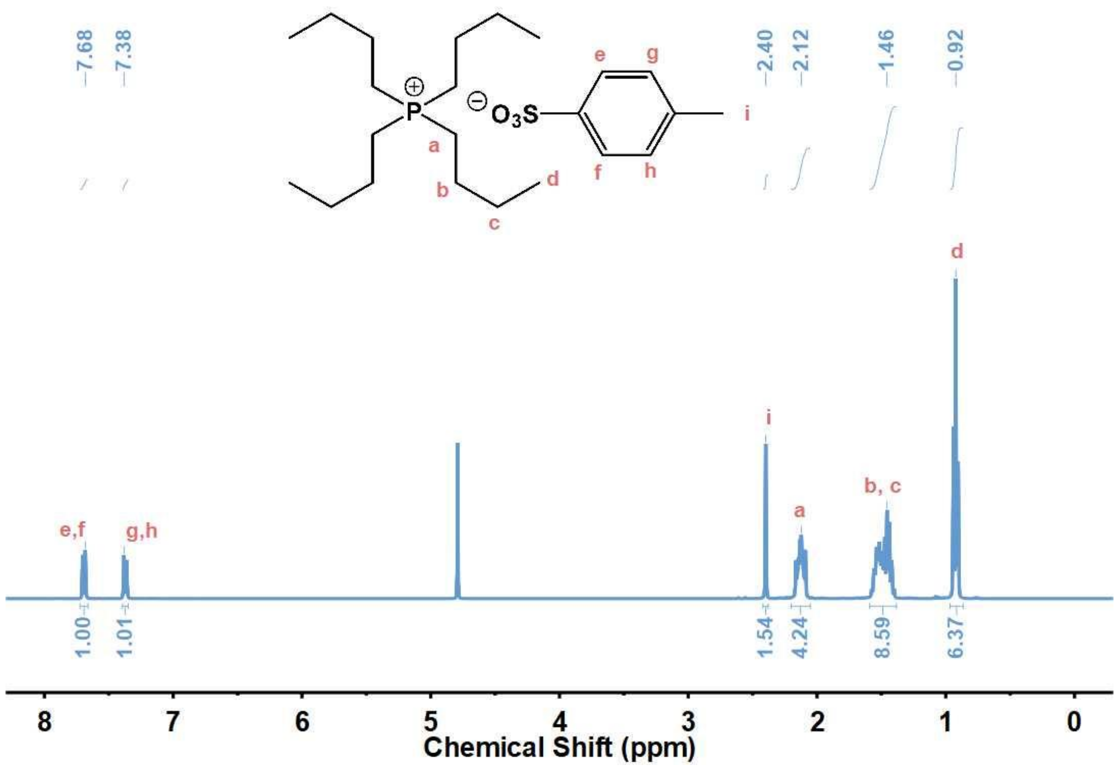


**Supplementary Fig. 5. $^{1}$H NMR spectra of [$P_{4444}$][TsO].**

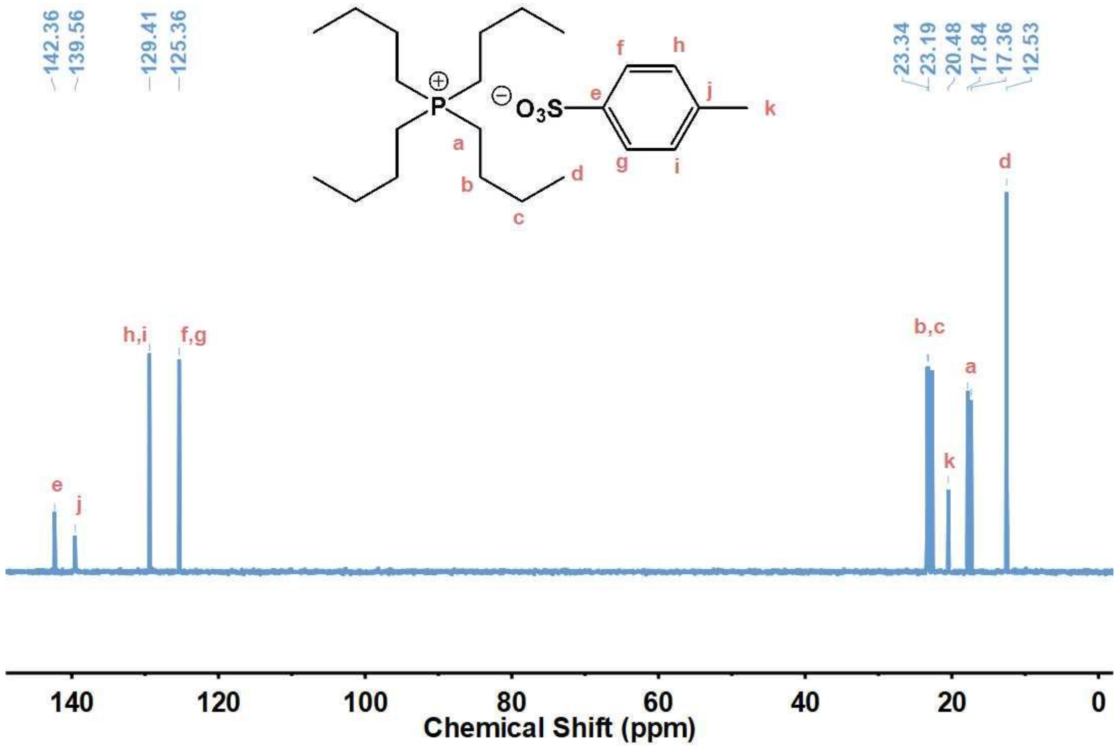


**Supplementary Fig. 6. $^{13}$C NMR spectra of [$P_{4444}$][TsO].**

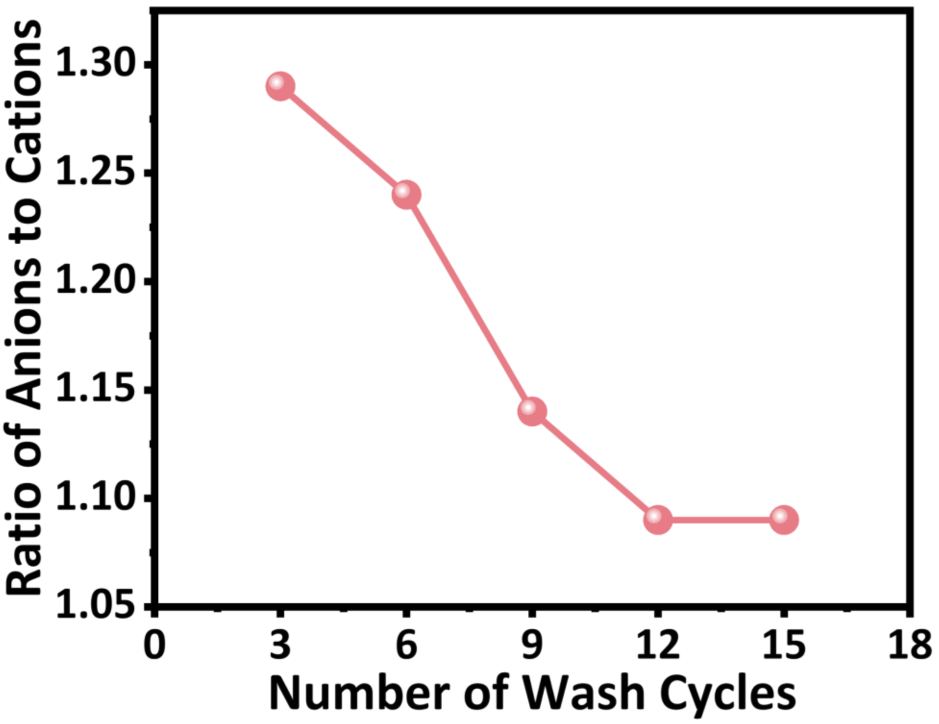


**Supplementary Fig. 7. Relationship between the number of wash cycles and the ratio of anions to cations in the synthesis process of [$P_{4444}$][TsO].**

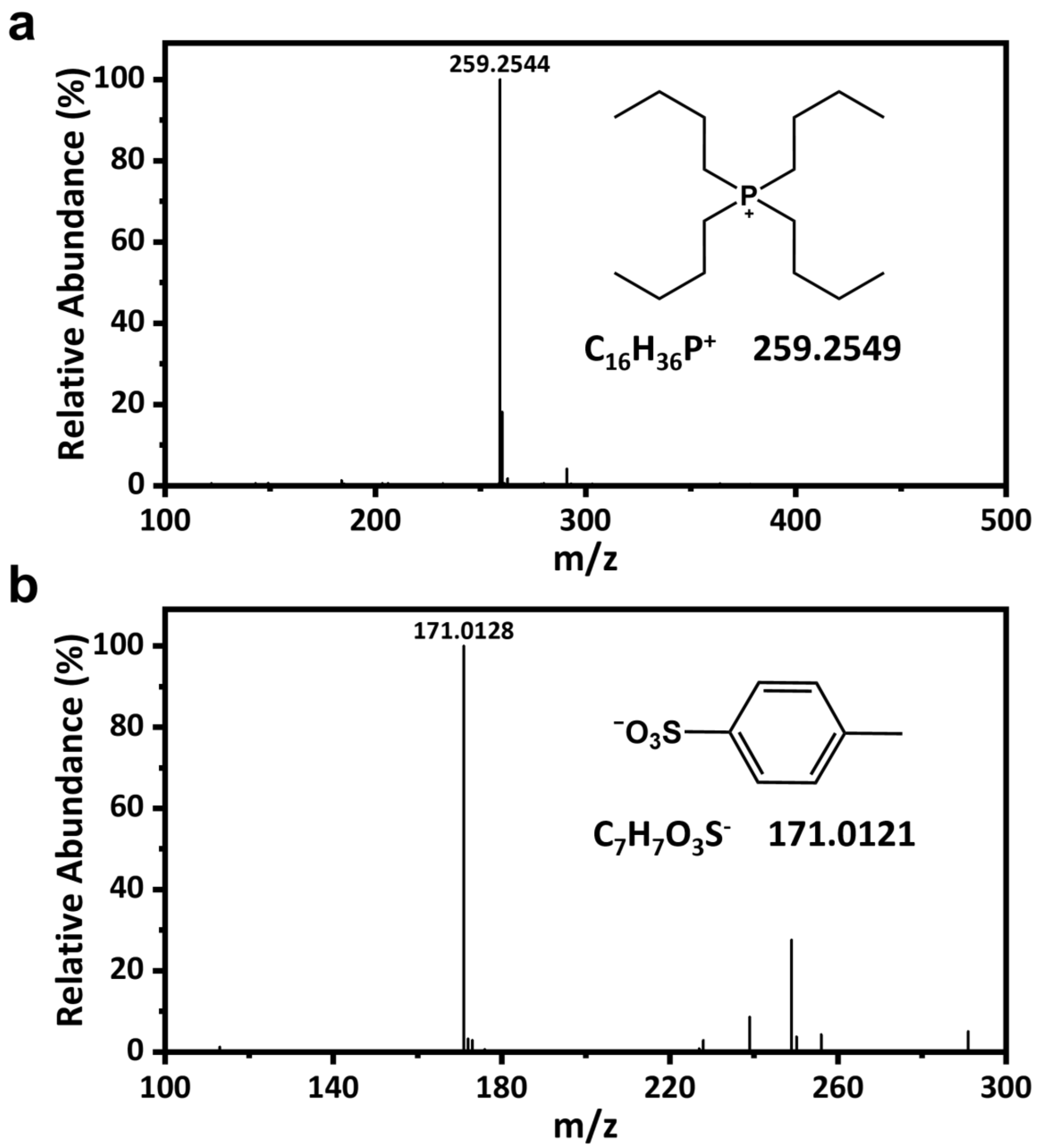


**Supplementary Fig. 8. Electrospray ionization mass spectrometry (ESI-MS) of [$P_{4444}$][TsO]. a** Positive mode; **b** Negative mode.

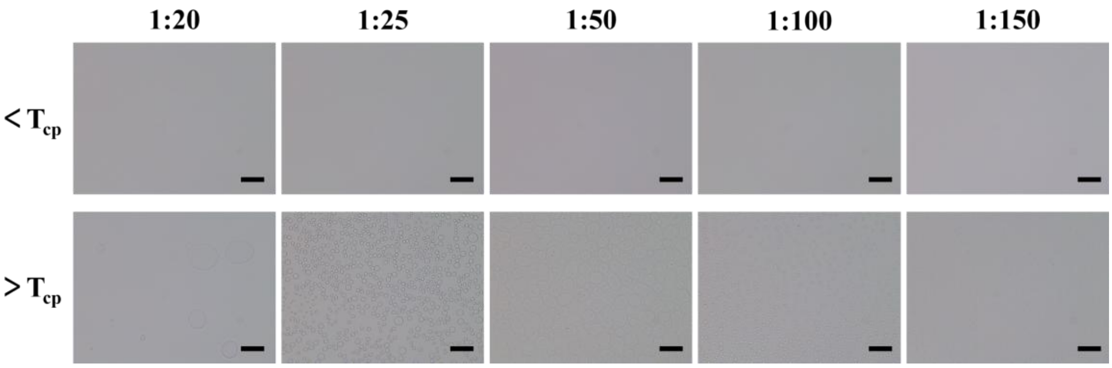


**Supplementary Fig. 9. Microscopic photographs of aqueous solutions of ionic liquids with different molar ratios before and after the phase transition.** Scale bars: 5.0 μm.

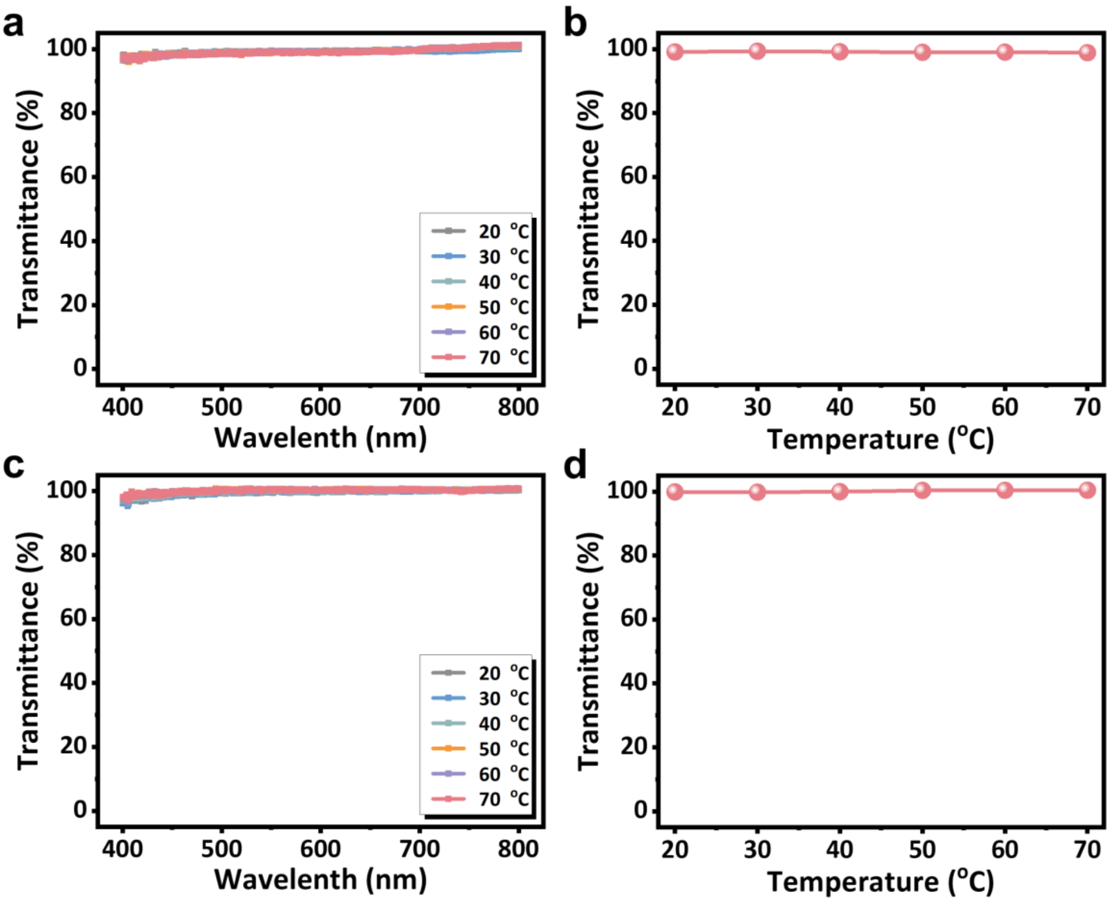


**Supplementary Fig. 10. Temperature-dependent UV-vis transmission spectra of [$P_{4444}$]Br and Na[TsO]. a-b,** [$P_{4444}$]Br; **c-d,** Na[TsO].

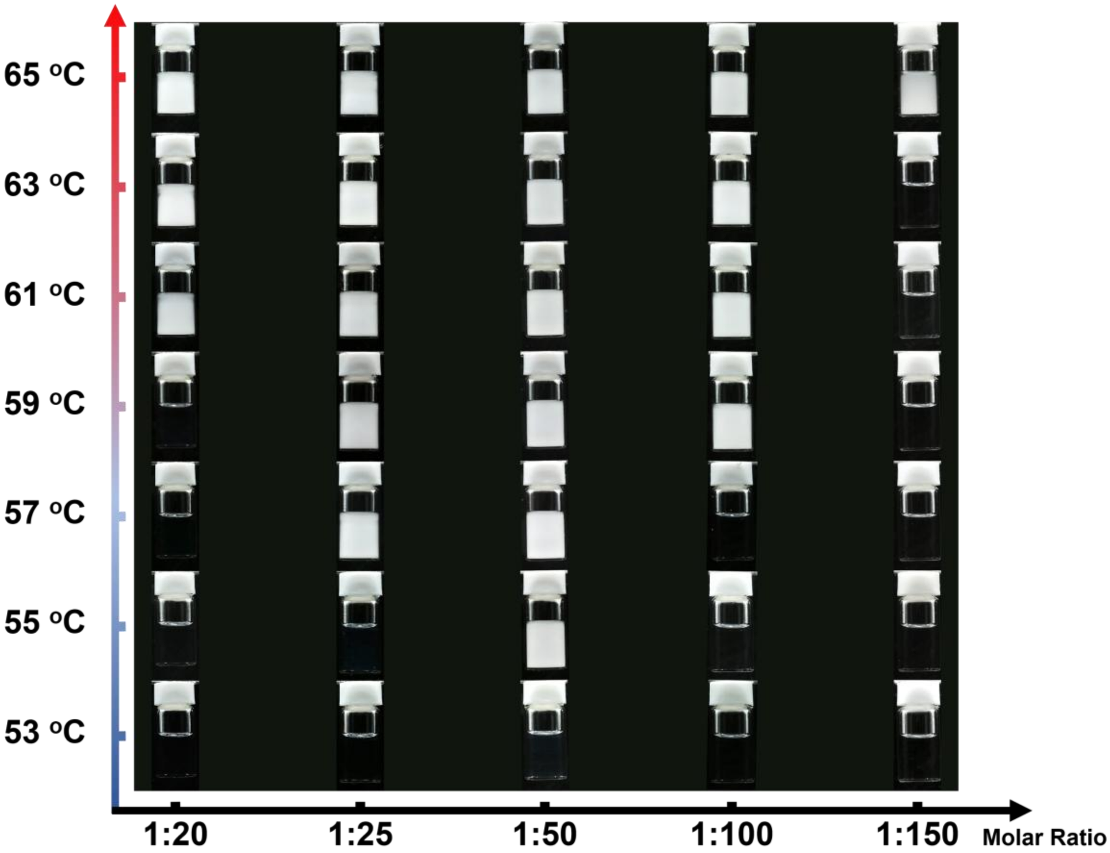


**Supplementary Fig. 11. Photographs of aqueous solutions of [$P_{4444}$][TsO] with different molar ratios.**

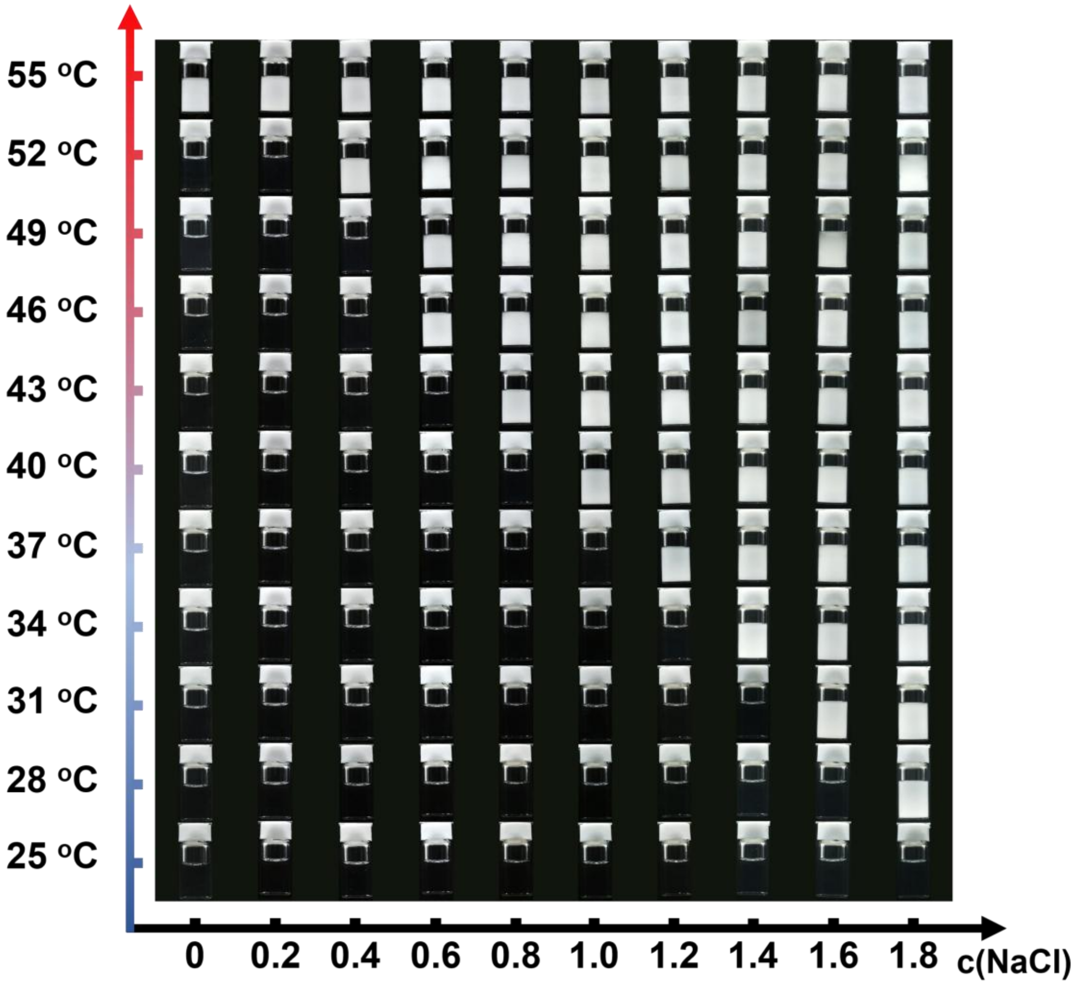


**Supplementary Fig. 12. Photographs of aqueous solutions of [$P_{4444}$][TsO] with a molar ratio of 1:50 by adding different concentrations of NaCl.**

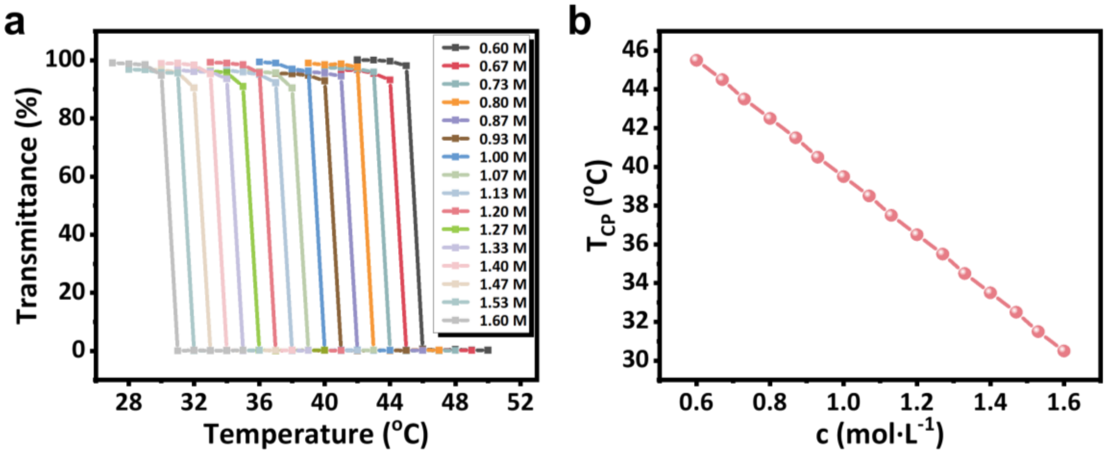


**Supplementary Fig. 13. Precise regulation of the 1 °C gradient in cloud point temperature ($T_{cp}$) of [$P_{4444}$][TsO] aqueous solutions.**

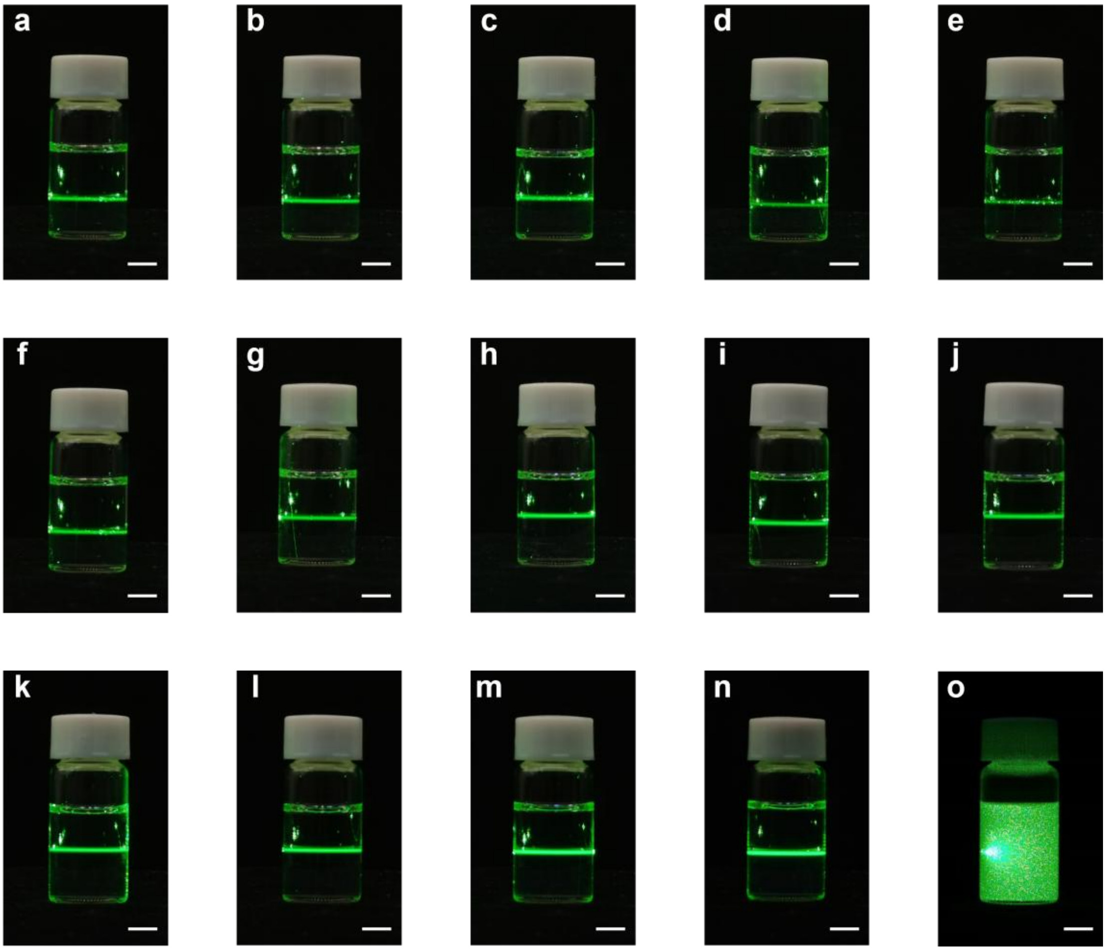


**Supplementary Fig. 14. Photographs of the Tyndall effect of [$P_{4444}$][TsO] solutions with different molar ratios at room temperature (from a to e) and [$P_{4444}$][TsO] solutions with a molar ratio of 1:50 with the addition of different concentrations of NaCl (from f to o). a,** 1:20; **b,** 1:25; **c,** 1:50; **d,** 1:100; **e,** 1:150; **f,** 0.2 M; **g,** 0.4 M; **h,** 0.6 M; **i,** 0.8 M; **j,** 1.0 M; **k,** 1.2 M; **l,** 1.4 M; **m,** 1.6 M; **n,** 1.8 M. Scale bars: 1.0 cm.

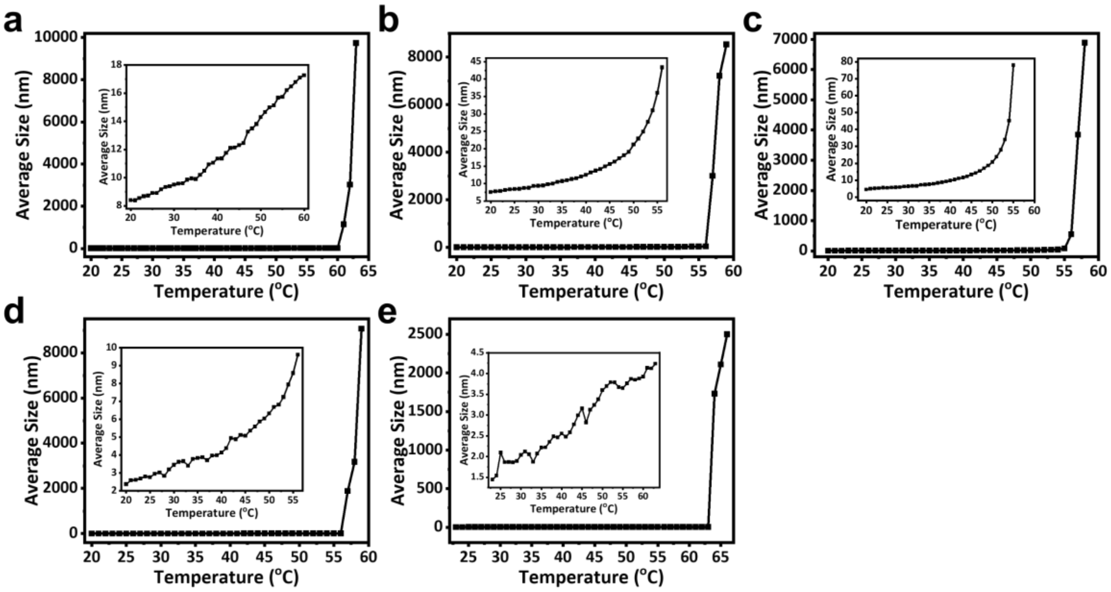


**Supplementary Fig. 15. Variation of the average hydration kinetic radius with temperature for aqueous solutions of [$P_{4444}$][TsO] with different molar ratios. a,** 1:20; **b,** 1:25; **c,** 1:50; **d,** 1:100; **e,** 1:150.

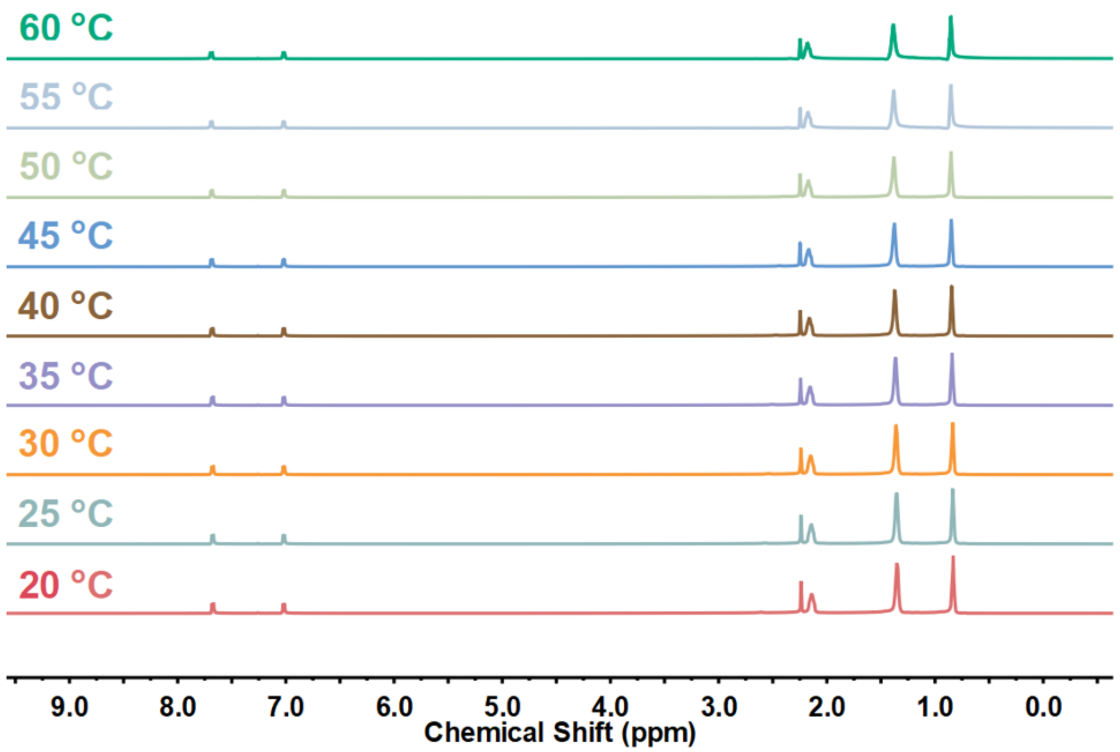


**Supplementary Fig. 16. Temperature-dependent $^{1}$H NMR spectra of [$P_{4444}$][TsO] in $CDCl_3$.** The temperature increased from 20 °C to 60 °C with an interval of 5 °C. Chemical shifts were not observed in this aprotic solvent.

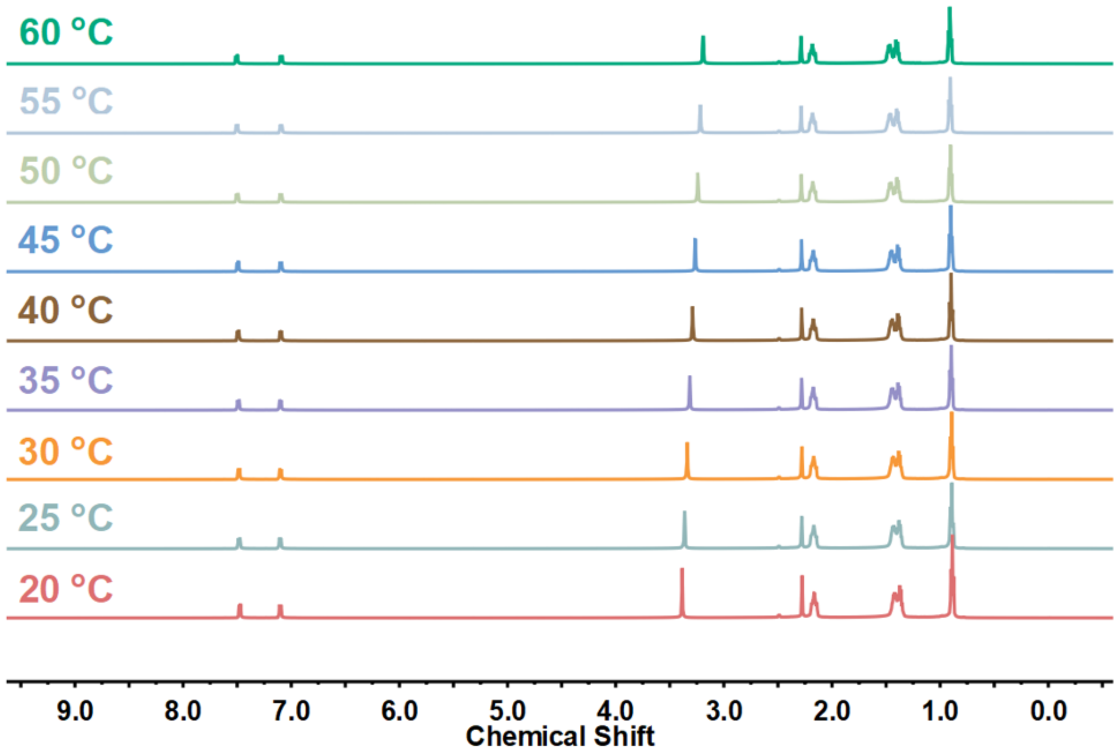


**Supplementary Fig. 17. Temperature-dependent $^{1}$H NMR spectra of [$P_{4444}$][TsO] in DMSO-d6.** The temperature increased from 20 ºC to 60 ºC with an interval of 5 ºC. The proton signal at 3.385 ppm corresponding to the remanent $H_2O$ under 20 ºC moved gradually to 3.193 ppm upon increasing the temperature to 60 ºC. Beyond that, each peak retained its own position as the temperature increased.

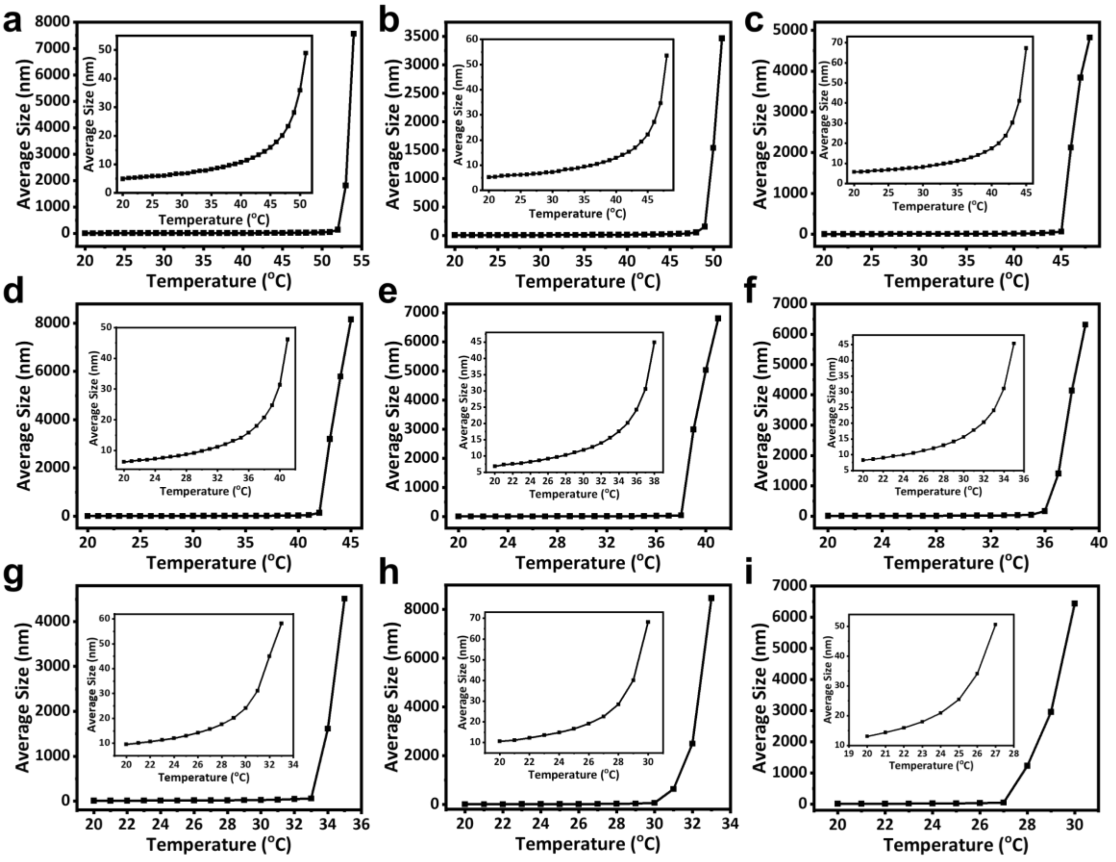


**Supplementary Fig. 18. Variation of mean hydration kinetic radius with temperature for aqueous solutions of [$P_{4444}$][TsO] with a molar ratio of 1:50 by addition of different concentrations of NaCl. a,** 0.2 M; **b,** 0.4 M; **c,** 0.6 M; **d,** 0.8 M; **e,** 1.0 M; **f,** 1.2 M; **g,** 1.4 M; **h,** 1.6 M; **i,** 1.8 M.

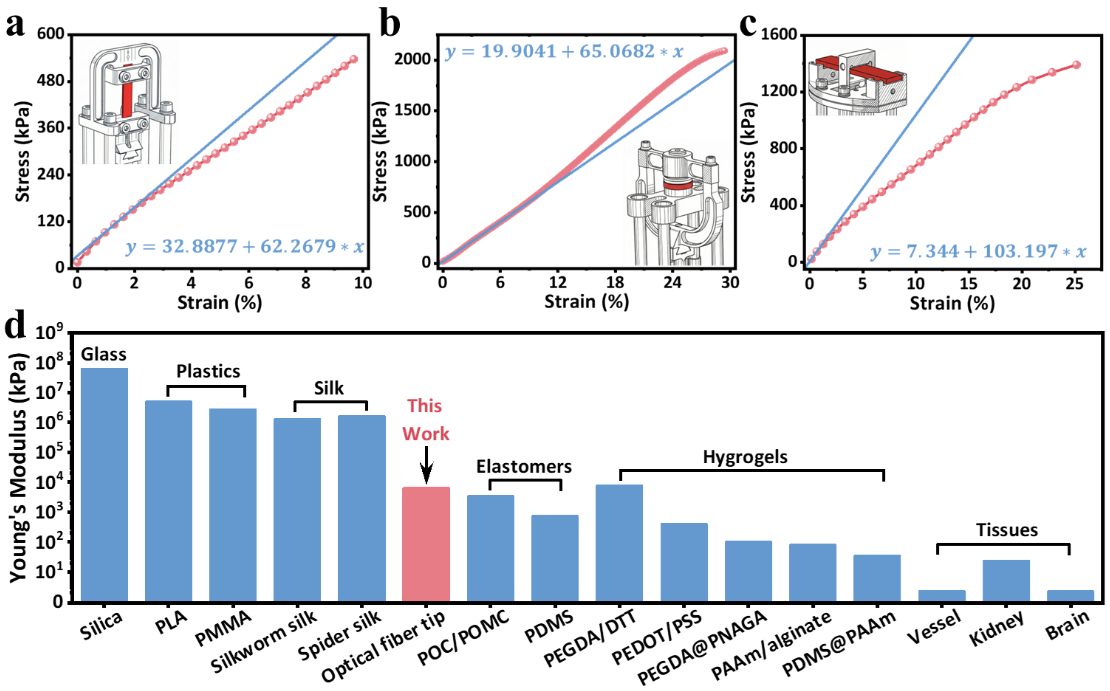


**Supplementary Fig. 19. Mechanical properties of the optical fiber tip. a**, Tensile modulus. **b,** Compression modulus. **c,** Bending modulus. **d,** Young's modulus of the optical fiber tip relative to other already published materials[4-18].

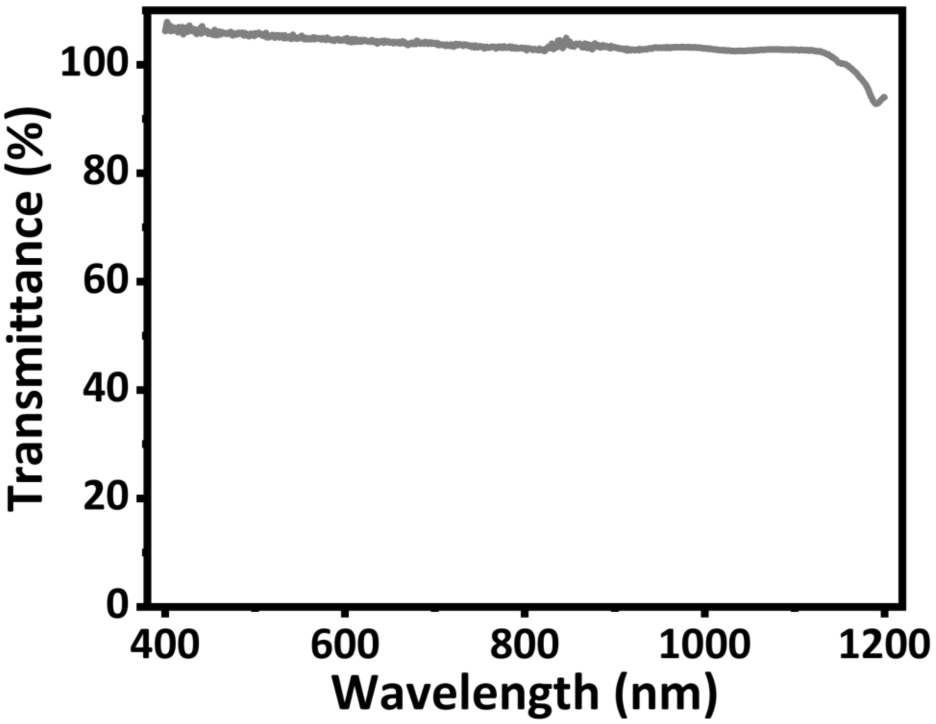


**Supplementary Fig. 20. UV-Vis absorption spectra of 1 mm thick nanotape.**

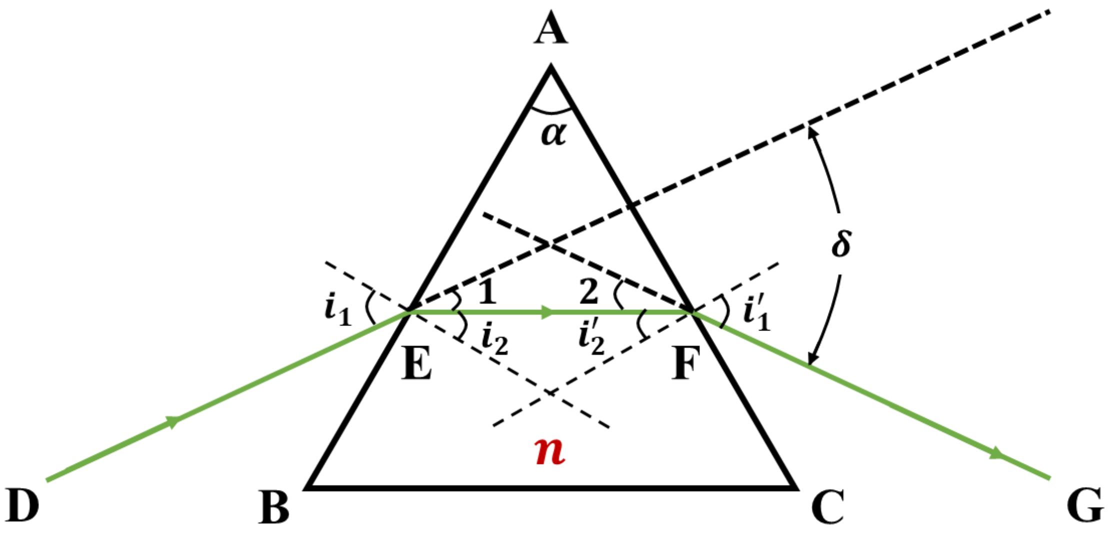


**Supplementary Fig. 21. Schematic diagram of the minimum deviation angle method for measuring the refractive index of liquids.**

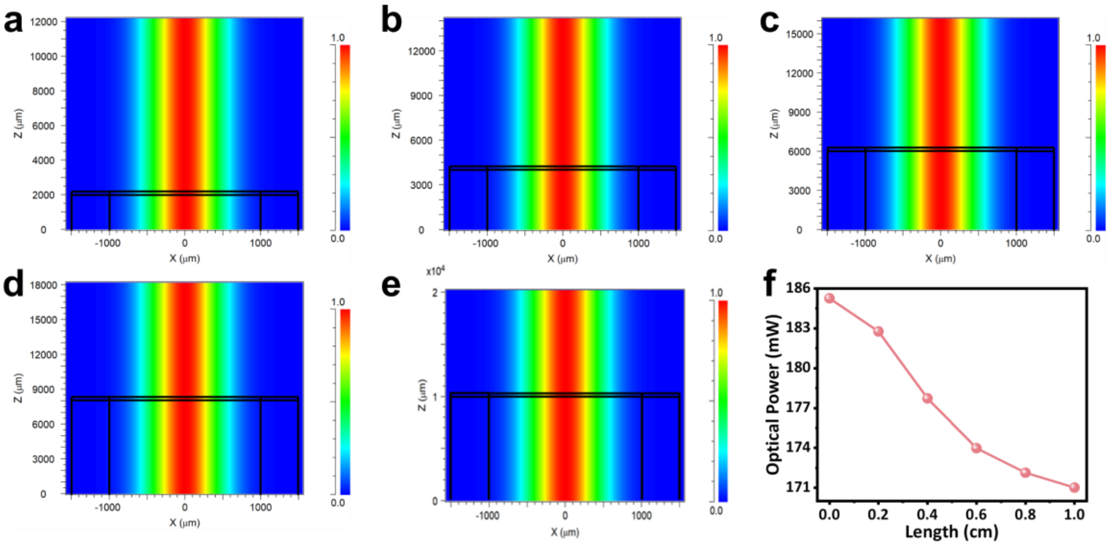


**Supplementary Fig. 22. Optical simulation and actual output optical power for different liquid column lengths. a,** 0.2 cm; **b,** 0.4 cm; **c,** 0.6 cm; **d,** 0.8 cm; **e,** 1.0 cm; **f,** Variation of optical power with liquid column length (n=6, mean±SD).

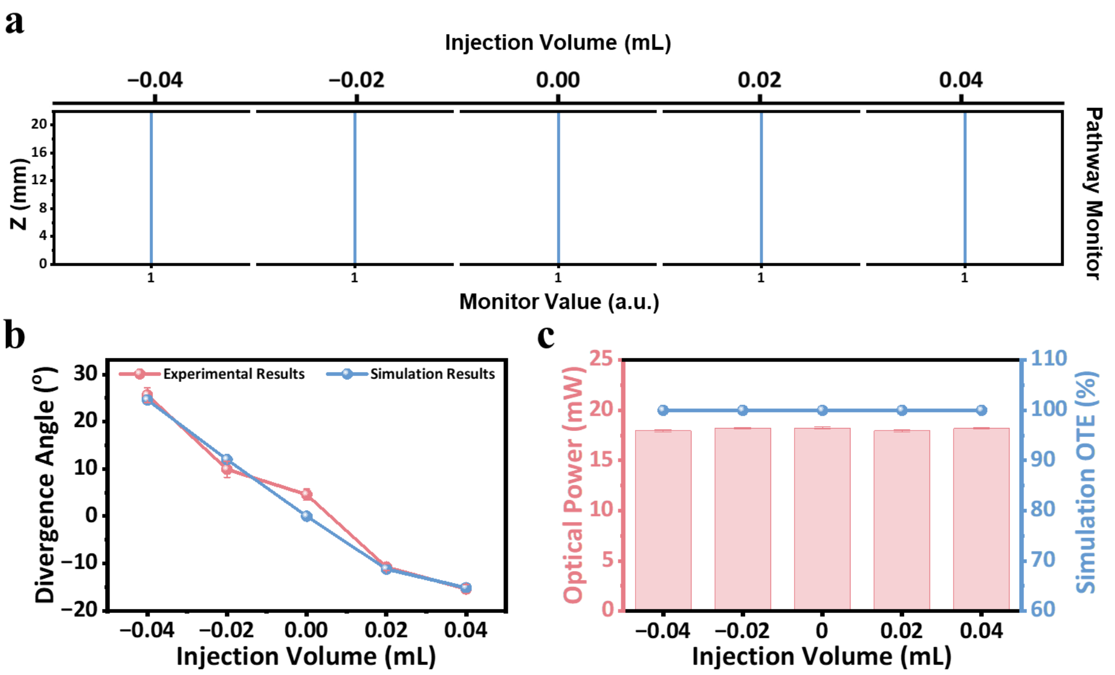


**Supplementary Fig. 23. Divergence angle regulation of AOWS at 532 nm. The experimental and simulation divergence angles under various outlet configurations.** **a,** Simulation of power changes during light transmission under various outlet configurations. **b,** The experimental and simulation divergence angles under various outlet configurations (n=5, mean±SD). **c,** The optical power and simulation optical transmission efficiency (OTE) under various outlet configurations (n=5, mean±SD).

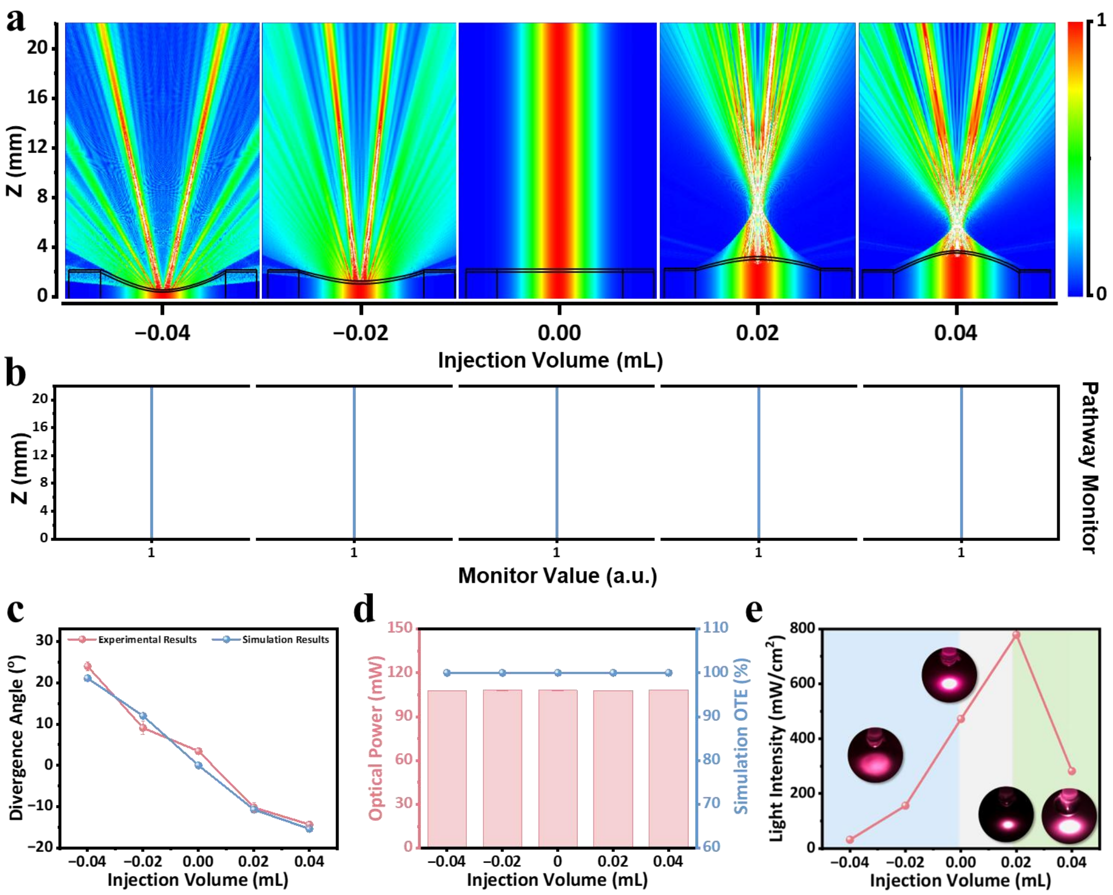


**Supplementary Fig. 24. Divergence angle regulation of AOWS at 808 nm. a,** Simulation of light exiting through five fiber outlets with different curvatures, according to the injection volume. **b,** Simulation of power changes during light transmission under various outlet configurations. **c,** The experimental and simulation divergence angles under various outlet configurations (n=5, mean±SD). **d,** The optical power and simulation optical transmission efficiency (OTE) under various outlet configurations (n=5, mean±SD). **e,** Light shedding area and power density of a parallel incident light exiting AOWS using optical fiber outlet with different features, either convex, flat or concave with varying curvatures (n=5, mean±SD).

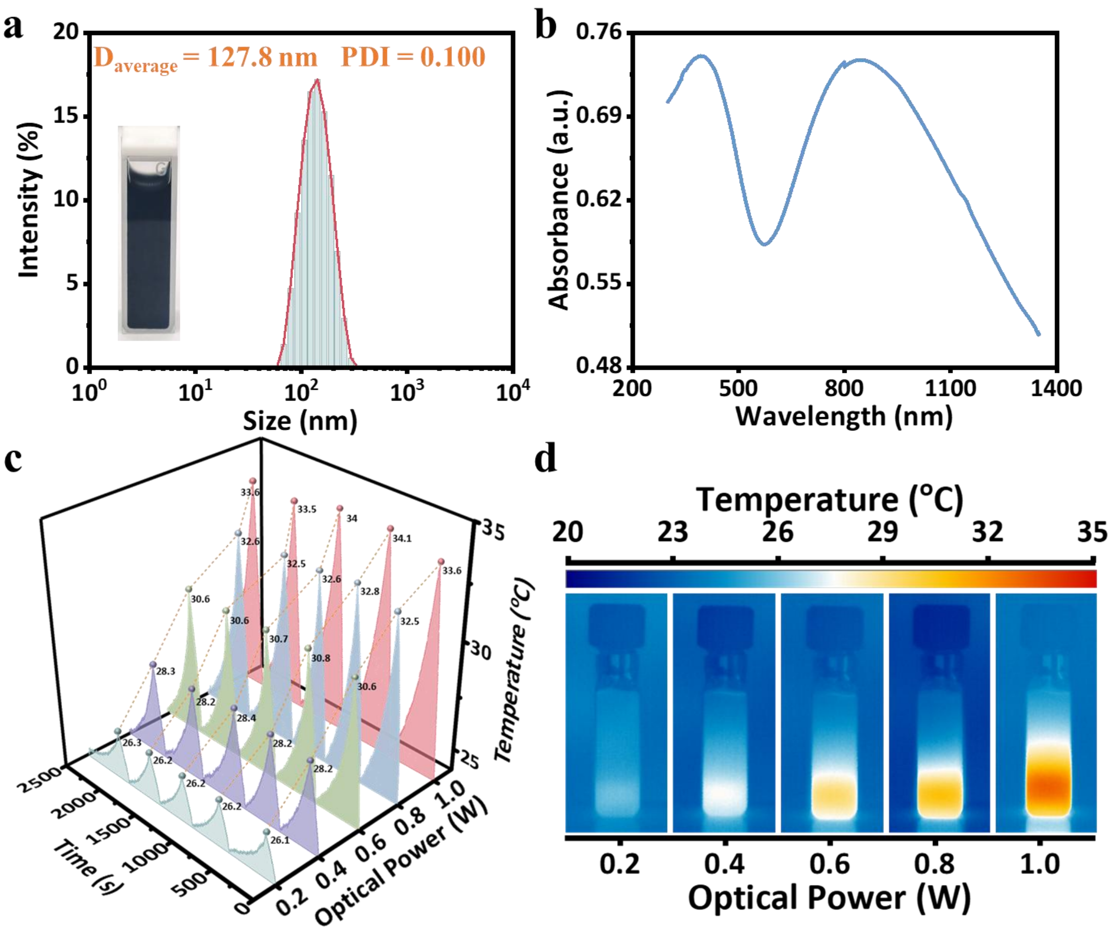


**Supplementary Fig. 25. Characterization of polypyrrole nanoparticles (PPy NPs).** **a,** Particle size of PPy NPs, inset is a photograph of PPy NPs particle dispersion. **b,** Absorption spectra of PPy NPs. Temperature rise of 0.08 mg/mL solution of PPy NPs under 808 nm laser irradiation with different powers **c** and infrared thermal imaging photographs **d**.

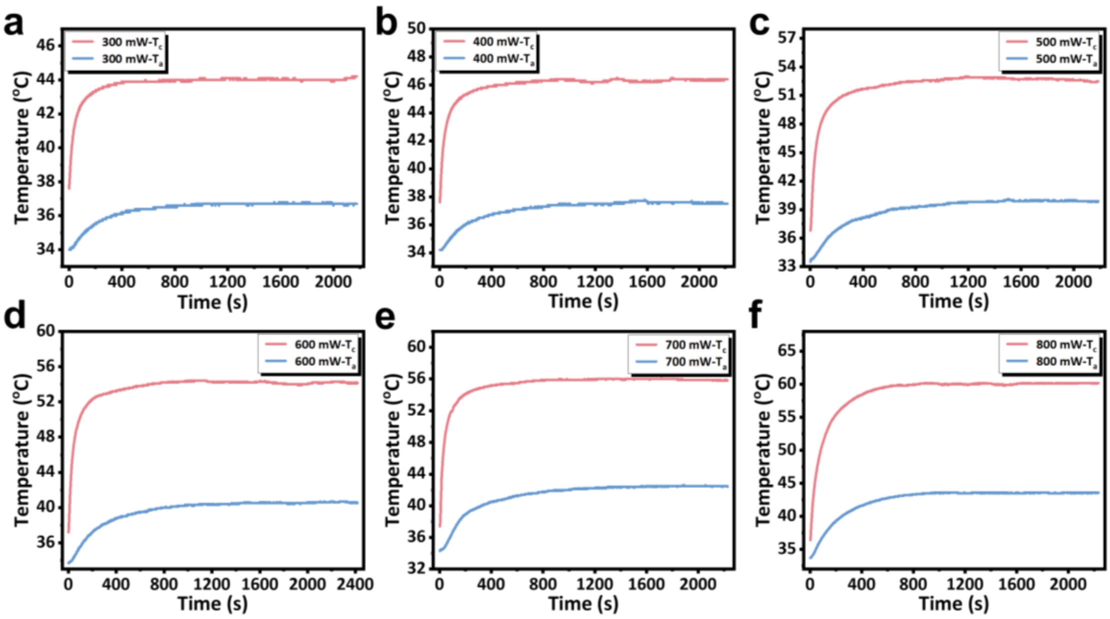


**Supplementary Fig. 26. Temperature rise of photothermal materials ($T_c$) and surrounding normal tissues ($T_a$) in NIR photothermal model.** It is worth noting that the tolerance power of skin to 808 nm laser is 330 mW/cm$^2$, which corresponds to an output power of 400 mW of the fiber in this case. Moreover, simulated tissues scatter less than skin, so the $T_c$ of NIR is difficult to meet the therapeutic demand in real-world application scenarios.

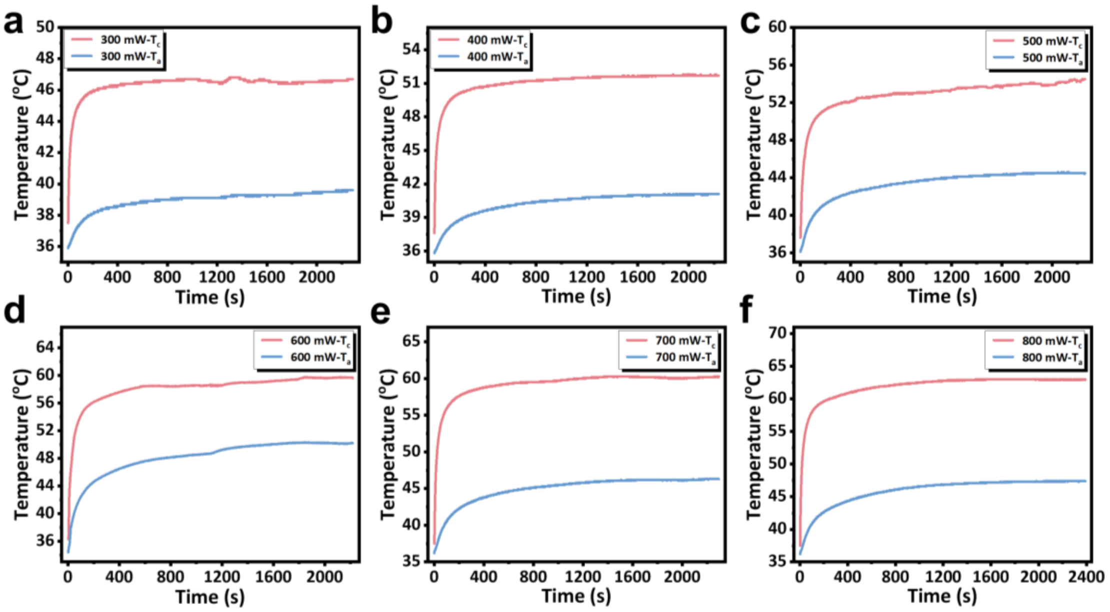


**Supplementary Fig. 27. Temperature rise of photothermal materials ($T_c$) and surrounding normal tissues ($T_a$) in OWS photothermal model.**

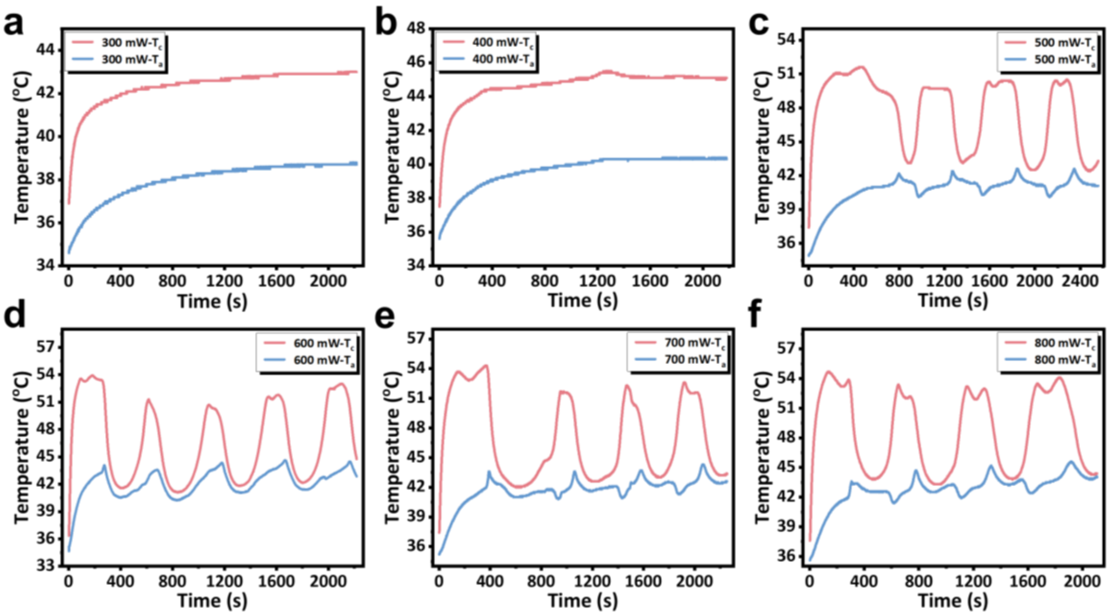


**Supplementary Fig. 28. Temperature rise of photothermal materials ($T_c$) and surrounding normal tissues ($T_a$) in AOWS photothermal model.**

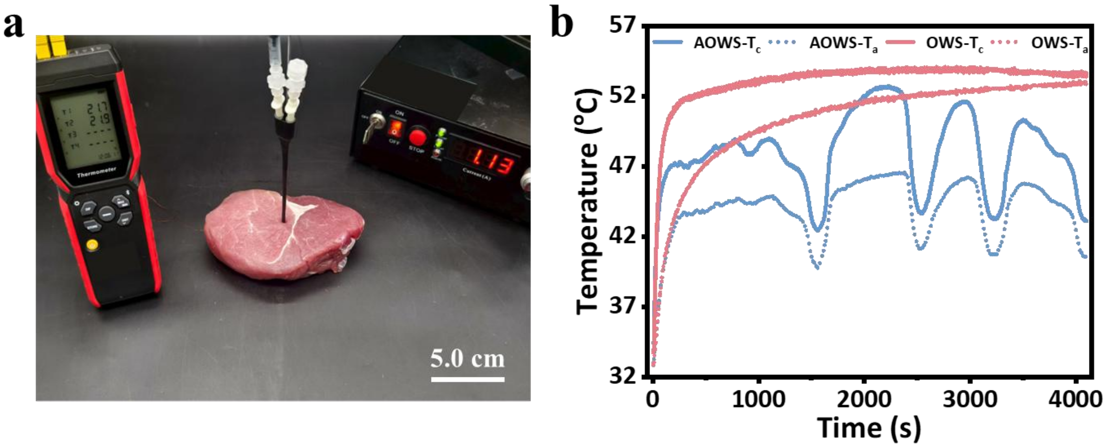


**Supplementary Fig. 29. Controllable photothermal performance of AOWS *ex vivo.***

**a,** Diagram of the setup for the isolated pork experiment. **b,** Temperature at the photothermal material ($T_c$) and the optical fiber tip ($T_a$) in AOWS and OWS experimental groups.

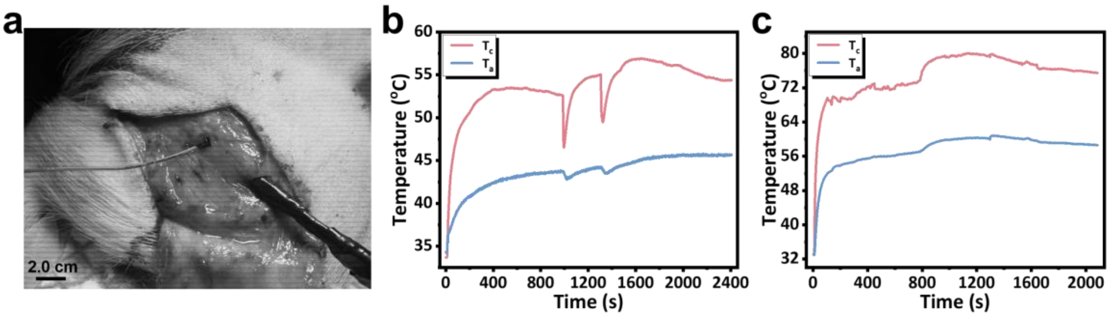


**Supplementary Fig. 30. Controllable photothermal performance of AOWS *in vivo*.** **a,** Photograph of the live pig photothermal experimental device; **b,** $T_a$ and $T_c$ in the experimental group implanted with AOWS; **c,** $T_a$ and $T_c$ in the control group implanted with OWS.

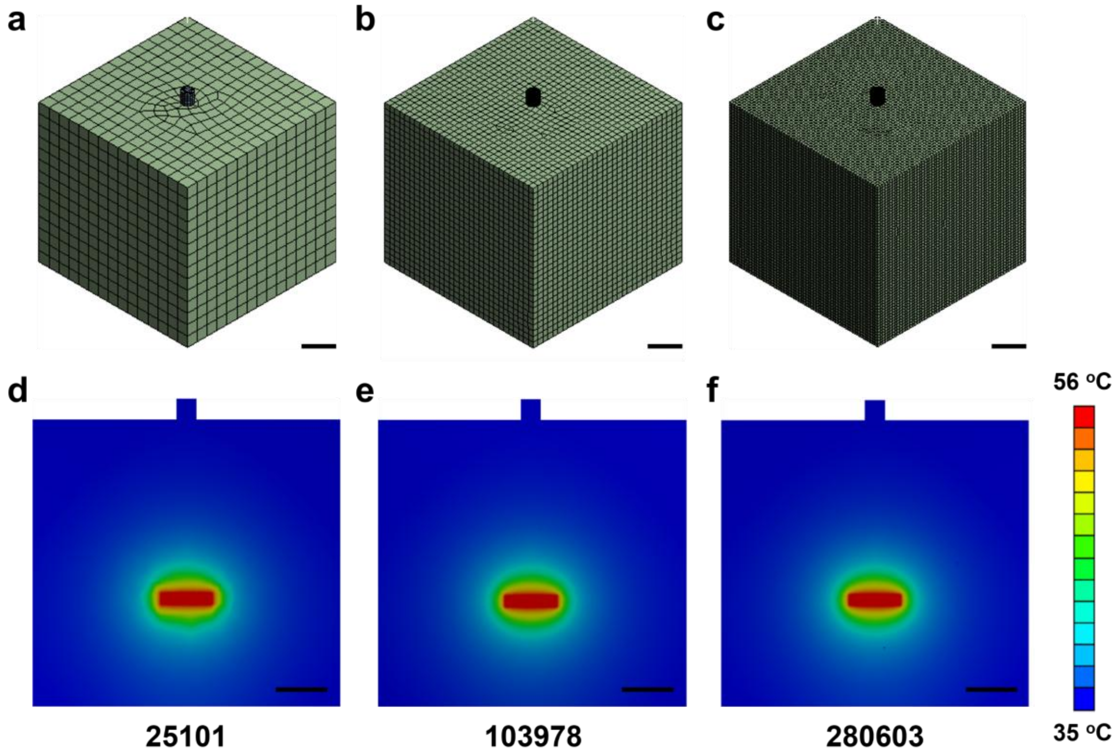


**Supplementary Fig. 31. The grid independence test for 3D temperature distribution simulation within abnormal tissues.** The cubic was divided into (**a** and **d**) 25101 units, (**b** and **e**) 103978 units and (**c** and **f**) 280603 units to perform the temperature distribution simulation. The consistent temperature distribution in **e** and **f** indicated the simulation was grid-independent. Scale bars: 5.0 mm.

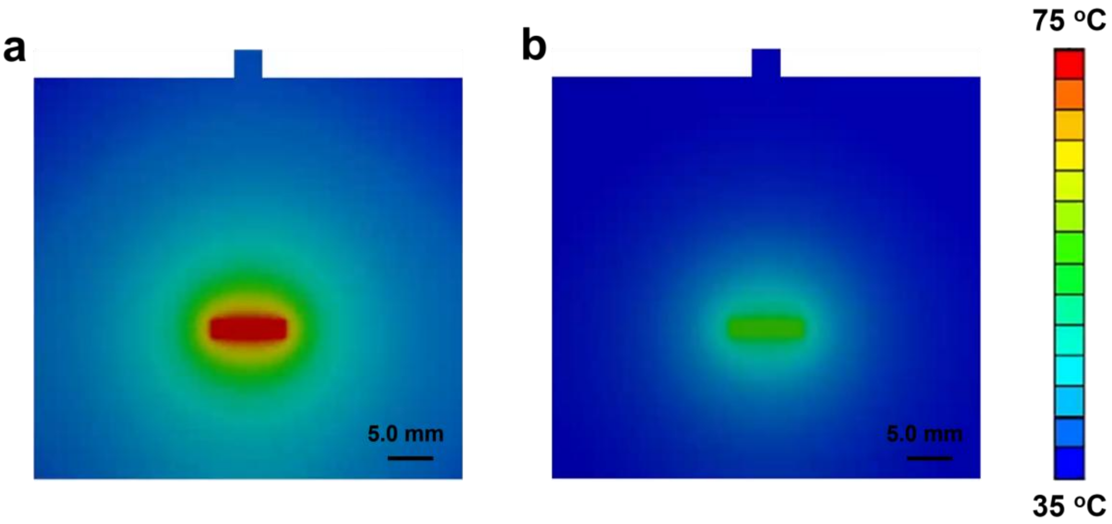


**Supplementary Fig. 32. Results of a 3D temperature distribution simulation of the temperature distribution within the abnormal tissue for a) OWS and b) AOWS.**

# 3. References for Supplementary Information


1. Cao, Y., Wang, Z., Liao, S. *et al*. A light-activated microheater for the remote control of enzymatic catalysis. *Chem. Eur. J. 22*, 1152 (2016).
2. Chen, M., Fang, X., Tang, S. Polypyrrolenanoparticles for high-performance in vivo near-infrared photothermal cancer therapy. *Chem. Commun.* **48**, 8934-8936 (2012).
3. Lee, J., Kim, S., Lee, Y. Measurement of Refractive Index of Liquids by the Maximum and Minimum Deviated Laser Beam. *Korean J. Opt. Photon.* **19**, 182–186 (2008).
4. Toland, K., Conway, A., Cunningham, L. *et al*. Development of a pulling machine to produce micron diameter fused silica fibres for use in prototype advanced gravitational wave detectors. *Class. Quantum Grav.* **35**, 165004 (2018).
5. Ilyas, R., Zuhri, M., Aisyah, H. *et al*. Natural fiber reinforced polylactic acid, polylactic acid blends and their composites for advanced applications. *Polymers* **14**, 202 (2022).
6. Yang, D., Yu, J., Tao, X. *et al*. Structural and mechanical properties of polymeric optical fiber. *Mater. Sci. Eng. A* **364**, 256–259 (2004).
7. Wang, M., Jin, H., Kaplan, D. *et al*. Mechanical properties of electrospun silk fibers. *Macromolecules* **37**, 6856-6864 (2004).
8. Agnarsson, I., Kuntner, M., Blackledge, T. Bioprospecting finds the toughest biological material: extraordinary silk from a giant riverine orb spider. *PLoS One* **5**,e11234 (2010).
9. Shan, D., Zhang, C., Kalaba, S. *et al*. Flexible biodegradable citrate-based polymeric step-index optical fiber. *Biomaterials* **143**, 142-148 (2017).
10. Guo, J., Niu, M., Yang, C. Highly flexible and stretchable optical strain sensing for human motion detection. *Optica* **4**, 1285-1288 (2017).
11. Feng, J., Zheng, Y., Bhusari, S. *et al*. Printed degradable optical waveguides for guiding light into tissue. *Adv. Funct. Mater.* **30**, 2004327 (2020).
12. Feig, V., Tran, H., Lee, M. *et al*. Mechanically tunable conductive interpenetrating

network hydrogels that mimic the elastic moduli of biological tissue. *Nat. Commun.* **9**, 2740 (2018)..

13. Zhu, B., Liu, D., Wu, J. *et al*. Slippery core-sheath hydrogel optical fiber built by catalytically triggered interface radical polymerization. *Adv. Funct. Mater.* **34**, 2309795 (2024).
14. Guo, J., Liu, X., Jiang, N. *et al*. Highly stretchable, strain sensing hydrogel optical fibers. *Adv. Mater.* **28**, 10244–10249 (2016).
15. Liu, B., Zhu, H., Zhao, D. *et al*. Hydrogel coating enabling mechanically friendly, step-index, functionalized optical fiber. *Adv. Opt. Mater.* **9**, 2101036 (2021).
16. Zhang, J., Zhou, J. The detection and evaluation of vascular stiffness. Acta Physiologica Sinica **74**, 894–902 (2022).
17. Radulescu, D., Peride, I., Petcu, L. *et al*. Supersonic shear wave ultrasonography for assessing tissue stiffness in native kidney. *Ultrasound Med. Biol.* **44**, 2556-2568 (2018).
18. Calhoun, M., Bentil, S., Elliott, E. *et al*. Beyond linear elastic modulus: viscoelastic models for brain and brain mimetic hydrogels. *ACS Biomater. Sci. Eng.* **5**, 3964−3973 (2019).
